# Trends in stroke outcomes in the last ten years in a European tertiary hospital

**DOI:** 10.1186/s12883-018-1164-7

**Published:** 2018-10-03

**Authors:** Emilio Rodríguez-Castro, Iria López-Dequit, María Santamaría-Cadavid, Susana Arias-Rivas, Manuel Rodríguez-Yáñez, José Manuel Pumar, Pablo Hervella, Esteban López-Arias, Andrés da Silva-Candal, Ana Estany, María Piñeiro-Lamas, Tomás Sobrino, Francisco Campos, Manuel Portela, Manuel Vázquez-Lima, José Castillo, Ramón Iglesias-Rey

**Affiliations:** 1 Clinical Neurosciences Research Laboratory, Health Research Institute of Santiago de Compostela (IDIS), Santiago de Compostela, Spain; 2 Stroke Unit, Department of Neurology, Hospital Clínico Universitario, Santiago de Compostela, Spain; 3 Department of Neuroradiology, Hospital Clínico Universitario, Santiago de Compostela, Spain; 4 Unit of Methodology of the Research, Health Research Institute of Santiago de Compostela (IDIS), Santiago de Compostela, Spain; 5 Consortium for Biomedical Research in Epidemiology and Public Health, Instituto de Salud Carlos III, Madrid. Health Research Institute of Santiago de Compostela, Santiago de Compostela, Spain; 6 Health Area Management of Santiago de Compostela, Servicio Galego de Saúde, Santiago de Compostela, Spain; 7 Emergency Department, Hospital do Salnés, Pontevedra, Vilagarcía de Arousa, Spain; 8 Clinical Neuroscience Research Laboratory (Hospital Clínico Universitario), Rúa Travesa da Choupana, s/n, 15706 Santiago de Compostela, Spain

**Keywords:** Ischemic stroke, Intracerebral hemorrhage, Mortality, Morbidity

## Abstract

**Background:**

Studying the impact of demographic changes and progress in the management of stroke patients is necessary in order to organize care structures for the coming years. Consequently, we analyzed the prognostic trends of patients admitted to the Stroke Unit of a tertiary hospital in the last ten years.

**Methods:**

The University Clinical Hospital of Santiago de Compostela is the referral hospital for stroke in a catchment area that accounts for 16.5% of the population of Galicia. Data from patients admitted to the Stroke Unit were registered prospectively. A multinomial logistic regression was performed to determine the influence of new trends in demographic factors and in the management of patients with acute stroke. For the expected trend of progression, a 2008–2011 and 2012–2017 time series model was made by selecting the most appropriate model.

**Results:**

In the last 10 years, the age of stroke onset has only increased in women (from 74.4 ± 2.2 years in 2008 to 78.8 ± 2.1 years in 2017; *p* = 0.037), and the same happens with the severity of neurological symptoms (ischemic stroke (IS), *p* < 0.0001; from 14 [10, 19] in 2008 to 19 [15, 26] in 2017), with a higher percentage of cardioembolic strokes (40.7% vs. 32.2% of cardioembolic strokes in women vs. men, *p* < 0.0001). In a multiple linear regression model, hospital improvement was mainly associated with the use of reperfusion treatment (B 53.11, CI 95% 49.87, 56.36, *p* < 0.0001). A differentiated multinomial logistic regression analysis conducted for the whole sample with ischemic strokes in the two time periods (2008–2011 and 2012–2017) showed no differences in the influence of factors associated with higher morbidity and mortality. The modeling of time series showed a distinct falling trend in mortality, with a slight increase in good outcome as well as morbidity in both ischemic and hemorrhagic stroke.

**Conclusions:**

Our results showed that mortality decreased in the entire sample; however, although outcome at discharge improved in ischemic stroke, severe disability also increased in these patients. Importantly, this tendency towards increased morbidity seems to be confirmed for the coming years.

## Background

In the last decades, there have been important changes in the management of acute stroke. These changes include modifications in the care chain of these patients [1–3], due to better diagnostic processes [4] and new therapeutic targets [5–8]. In the same line, other strategies as the incorporation of multidisciplinary and specialized Stroke Units (24-h neurology and interventional neuroradiology attention) [9], the protocolization of hemodynamic [10], thrombolytic [11] and endovascular recanalization treatments [12], as well as the creation of specific neurology wards and telemedicine systems [13] have provided improvements in patient care. This situation has led to a new paradigm in the appreciation of the emergency standard of care and to an improved knowledge of the patient with stroke, both in healthcare structures and by the public at large [14].

The new healthcare scenario has been accompanied by more effective and more generalized primary prevention measures [15, 16]. However, in a context of deep demographic changes, the age-standardized incidence of stroke in Europe and developed countries is increasing and it seems that it will continue to do so in the next decades [17–19].

Galicia is a Spanish region on the northwest of the Iberian Peninsula, with a total population of 2,707,700 inhabitants concentrated mainly on the coastal areas (population density of 92.5 inhabitants/km^2^). Life expectancy at birth is 82.6 years and has increased by 5 years since 1981.

The percentage of aging (population over the age of 65 divided by population younger than 15 multiplied by 100) of Galicia is 151.9%. In the public network hospitals of Galicia, stroke admissions account for 2.35% of the total hospitalization. This rate has remained stable for the last five years and is similar for both sexes [20].

Therefore, the analysis of the impact of all these changes on the clinical results obtained should provide valuable data to further organize health system structures and improve management protocols. For this purpose, we analyzed the trend in the progress of patients admitted to a specialized Stroke Unit of a tertiary hospital (Galicia) in the last ten years.

## Methods

### Study population and patient characteristics

**Fig. 1:**
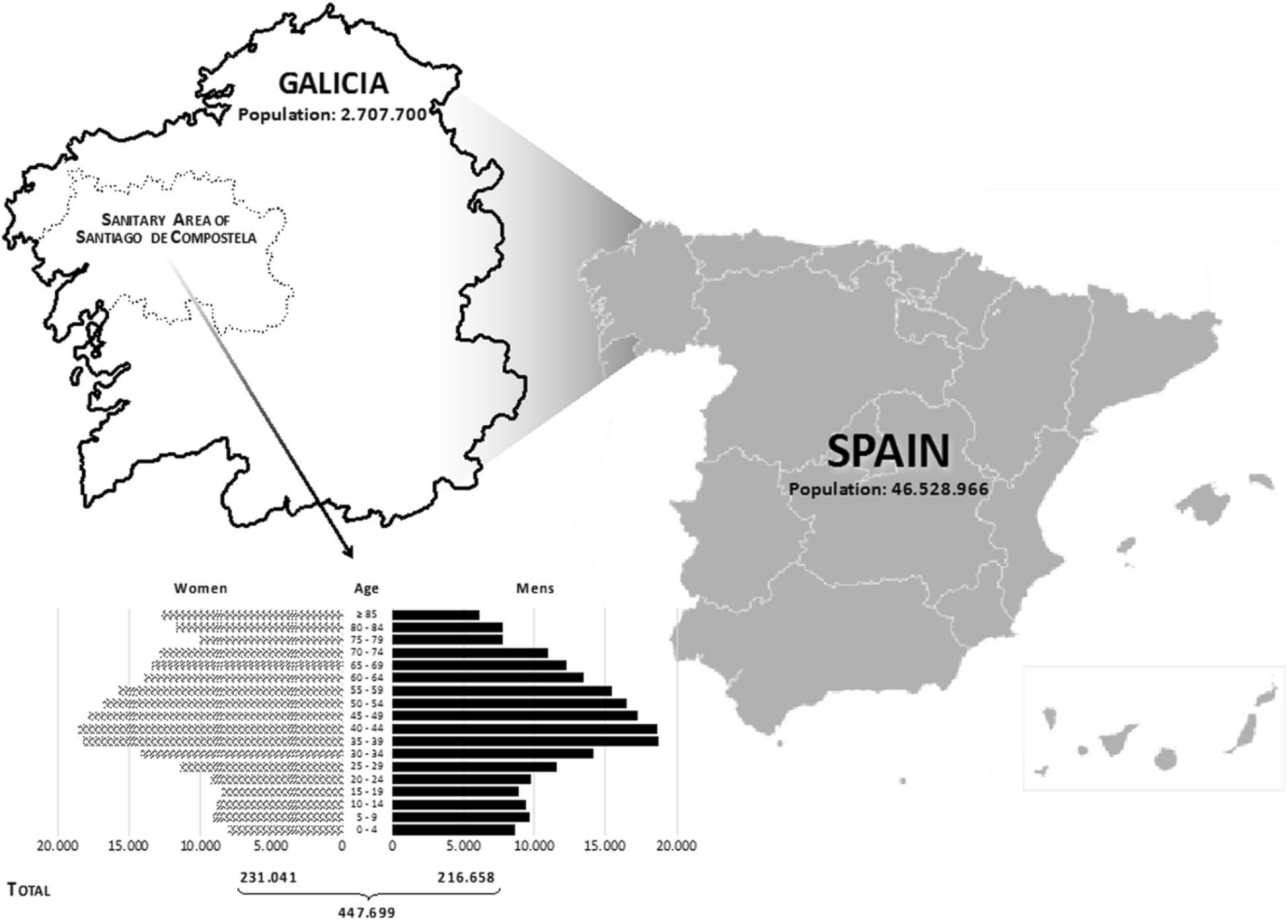
Demographic data of the Health Area of Santiago de Compostela. [Instituto Galego de Estadística, 2016. Instituto Nacional de Estadística, 2016 (www.ige.eu)]

The University Clinical Hospital of Santiago de Compostela serves a catchment area of 447,699 people (16.5% of the total Galician population), plus 18–22% referred from other health centers. The center has a Stroke Unit and provides 24-h neurology and interventional neuroradiology attention (Fig. 1).

Since October 2004, we have prospectively included patients with ischemic stroke (IS) and non-traumatic intracerebral hemorrhage (ICH) admitted to the Stroke Unit of the University Clinical Hospital of Santiago de Compostela in the BICHUS registry. We would like to note that the present study was conducted using the last 10-year data (From September 2007 to September 2017). This research was conducted in accordance with the Declaration of Helsinki of the World Medical Association (2008) and approved by the Ethics Committee of Galicia (EC). Written informed consent was obtained from each patient or from their relatives after full discussion of the procedures.

### Clinical variables

The variables of our registry included stroke date, latency time from the stroke onset and care in the Emergency Department, and whether it was a wake-up stroke. Demographic data, previous modified Rankin scale (mRS) [21] and vascular risk factors were included. Data of previous carotid disease and processes of carotid revascularization were also collected, and in the case of patients with a previous transient ischemic attack (TIA), the time between TIA and stroke, as well as the coincidence or not with topography.

The clinical variables collected were: National Institute of Health Stroke Scale (NIHSS) [22] on admission, at 48 h and at discharge, mRS at discharge and at 3 months, and maximum axillary temperature within the first 24 h.

Blood sample measurements were: glucose levels at admission, glycated hemoglobin, white blood cell, red blood cell and platelets count, fibrinogen, C-reactive protein, total and fractionated cholesterol, triglycerides, proBNP, D-vitamin, and cholecalciferol. Neuroimaging variables in ischemic stroke were: baseline infarct volume (DWI-lesion) and final ischemic lesion volume (a second CT between 4^th^–7^th^ day) and, existence and type of hemorrhagic transformation. In hemorrhagic stroke we collected basal hematoma volume (CT) and hematoma and edema volume (second CT between 4^th^–7^th^ day). We evaluated the neurological progress during hospitalization according to the following formula: (NIHSS on admission - NIHSS on discharge) / NIHSS on admission) × 100. Patients who died during hospitalization were given a zero score. The mRS at 3 ± 1 months was assessed face-to-face in 3151 patients and by telephone in 1731. Accredited expert neurologists assessed both scales (ER-C, IL-D, MS-C, SA-R, MR-Y). The etiological diagnosis of IS was performed according to TOAST criteria [23]. Non-traumatic ICH was classified into hypertensive, amyloid, related to antiplatelets / anticoagulants and undetermined [24, 25]. We also recorded whether patients were treated by telemedicine, type of reperfusion therapy and complications during the acute phase.

Clinical data were available of all patients, laboratory variables in 87% of cases and neuroimaging variables in 73% of patients.

### Statistical analysis

From September 2007 to September 2017; 6129 patients were included. Patients who: suffered from transient ischemic attack (512), were transferred to other hospital during the acute phase (42), died for causes other than vascular (39), had non-confirmed stroke diagnosis (85), were referred from other hospital after acute phase management (94) and were lost to follow-up at 3 months (475) were excluded. Finally, 4882 patients (3921 with IS and 961 with ICH) were considered valid for the analysis.

Results were expressed as percentages, mean and standard deviation or median and 25–75% percentiles, and the differences were determined by the chi-square, t-student or Mann-Whitney-Wilcoxon tests. The distribution of the same variable in the 10 years of the study was analyzed using ANOVA test. The relationship between the two variables was determined by Pearson or Spearman coefficients, depending on the nature of the variable.

In 2012, endovascular treatment was implemented as the standard of care in our hospital, so we compared 2008–2011 and 2012–2017 periods to determine the influence of new trends in the management of patients with acute stroke. First, a bivariate multinomial logistic regression (MLR) was performed, testing the significance of each of the variables considered as candidates from a clinical approach. Next, a multivariate analysis was proposed, using the MLR technique. At this point, the variables whose *p*-values of the likelihood ratio contrast were > 0.20 were discarded. Strategy was repeated until finding the definitive set of variables that integrated all those that were significant (*p* < 0.05) for the variable mRS at 3 months. Three models were constructed following this methodology and differentiating IS from ICH: using all data (model 1), using data from the 2008–2011 period (model 2) and using data from the 2012–2017 period (model 3). To determine the influence of variables in the in-hospital improvement we performed a multiple linear regression model adjusted for those variables that reached statistical significance *p* < 0.05 in univariate analysis [IBM SPSS Statistics software v19.0].

The expected trend in patient progress for the next few years at 3 months after a stroke – categorized as good outcome (mRS ≤ 2), morbidity (mRS > 2 and ≤ 5) and mortality (mRS = 6) – was estimated by time series model. A time series trend model was made by selecting the most appropriate one (from a list of 5 possible models) through Schwarz Information Criteria (SBIC) and Akaike Information Criteria (AIC). Once the time series trend was estimated, the residual component was analyzed. To determine whether the residual series was white noise or not, graphic tests, the tests of Ljung-Box and Durbin-Watson were performed. In the cases where the residual series were not white noise, this residual component was modeled through autoregressive and moving averages models (AR, MA or ARMA). Once the models are adjusted, the forecasts are calculated as the sum of the forecasts of both components. In the cases where the residual series are white noise, their forecast will be zero [R statistical software v3.2.21].

## Results

### Demographic, clinical and progress characteristics of the sample analyzed

**Fig. 2:**
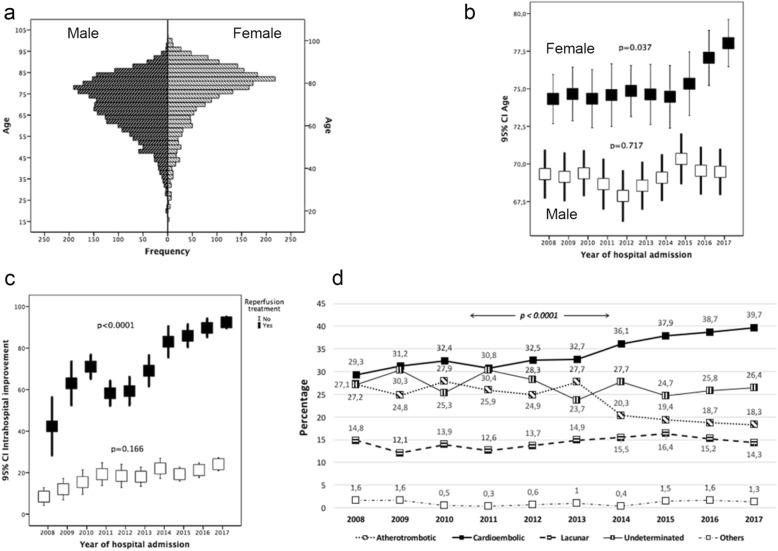
Demographic, clinical and progress characteristics of the sample analyzed: **a** Age pyramid of the sample analyzed by sex. **b** Trends in the distribution by age and sex. **c** Trends in intrahospital progress according to the reperfusion treatment. **d** Trends in the distribution by etiopathogenesis (ischemic stroke)

The mean age was 71.9 ± 13.9 years; 55.3% were male and 44.7% female. The pyramid of age and sex distribution can be seen in Fig. 2a (80.3% IS and 19.7% ICH). The IS subtypes were classified as: 23.6% atherothrombotic, 36.5% cardioembolic, 7.9% lacunar, 30.9% indeterminate and 1.1% of rare causes. Of ICH, 48.9% were hypertensive, 6.7% amyloid, 13.6% antiplatelet/anticoagulant, and 30.8% undetermined (26.7%) or due to unusual causes (4.1%).

8.9% of patients were wake-up stroke (9.9% of IS and 4.7% of ICH), and in the rest, the latency time between the symptoms onset and hospital arrival was 218.9 ± 177.2 min (219.8 ± 168.1 min in IS and 215.0 ± 210.8 min in the ICH). 1.6% of patients were treated in another hospital by a telemedicine system.

Of the whole sample analyzed, 62.5% of patients presented with hypertension, 23.4% had diabetes, 15.6% were smokers, 12.5% had alcoholic habit, 34.6% hyperlipidemia, 18.8% atrial fibrillation, 16.4% carotid disease (13.4% ipsilateral, 1.4% contralateral and 1.6% bilateral) and 4.5% had suffered a previous transitory ischemic attack, 3 ± 4.6 days before. The NIHSS score on admission was 13 [8, 19] and percentage of hospital improvement was 28.9 ± 44.7%. At 3 months, 52.2% had a good outcome and mortality was 15.6%.

Since 2015, the age of stroke patients admitted has shown a progressive increase, especially significant in women (Fig. 2b). The percentage of wake-up stroke did not change in the 10-year analyzed (10.7 vs. 9.8%, *p* = 0.071), and there was a significant reduction in time between stroke onset and hospital arrival (269.8 ± 225.5 min in 2008 and 202.1 ± 201.3 min in 2017, *p* < 0.0001).

The severity of neurological symptoms determined by NIHSS on admission has increased progressively (*p* < 0.0001) in patients with IS (14 [10, 19] in 2008 to 19 [15, 26] in 2017), and has remained stable (*p* = 0.176) in ICH (14 [9, 18] to 15 [8, 22]). Hospital improvement has shown a marked increase since 2009 in IS (*p* < 0.0001) and has remained stable (*p* = 0.302) in ICH. The improvement in IS was associated with the increase in reperfusion therapies, from 9.1% in 2008 to 28.8% in 2107 (Fig. 2c).

In the years under analysis, the trend in the global distribution of the IS and the ICH has remained stable (*p* = 0.602). However, among the IS there has been an increase in cardioembolic stroke origin (29.3% to 39.7% in 10 years). Patients with cardioembolic stroke were older (age in atherothrombotic 69.3 ± 13.7, cardioembolic 75.2 ± 13.3, lacunar 67.1 ± 13.4, undetermined 70.7 ± 14.2, unusual causes 56.5 ± 16.2 years, *p* < 0.0001) and tended to be women (40.7% vs. 32.2% of cardioembolic strokes in women vs. men, *p* < 0.0001) (Fig. 2d). The etiology of the ICH has remained stable (*p* = 0.286).

In a multiple linear regression model, hospital improvement was mainly associated with the use of reperfusion therapy (B 53.11, CI 95% 49.87, 56.36, *p* < 0.0001), but a positive trend in patient progress has also been found in the last ten years (B 0.88, 95% CI 0.32, 1.43, *p* = 0.002).

### Trends in the development of the incidence of factors that modify the risk of stroke

**Fig. 3:**
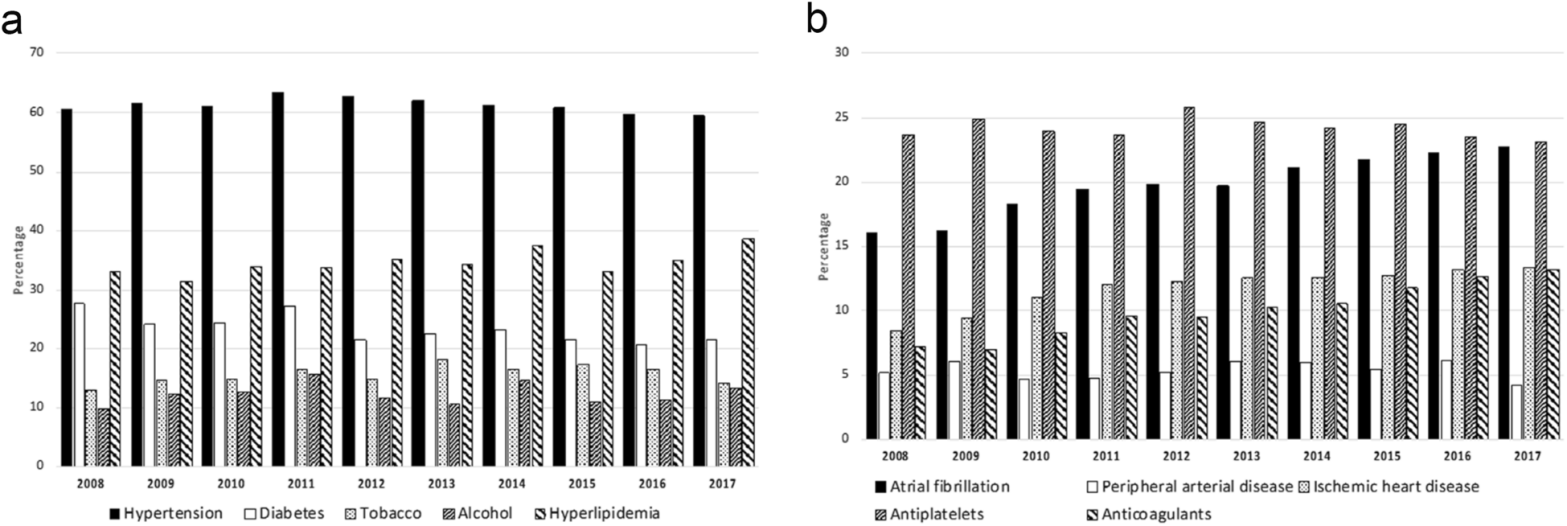
Trends of the factors that modify the risk of stroke: **a** Classic vascular risk factors. **b** Factors of heart disease, peripheral arterial disease and preventive drugs

Classical risk factors, such as arterial hypertension (*p* = 0.648), tobacco (*p* = 0.931), alcohol use (*p* = 0.550) and hyperlipidemia (*p* = 0.193), showed no variation in the last ten years. The incidence of diabetes has decreased (*p* = 0.036) from 27.6% in 2008 to 21.5% in 2017 (Fig. 3a). Fig. 3b shows the progress of heart diseases, peripheral arterial disease and the administration of preventive drugs. The incidence of atrial fibrillation (*p* < 0.0001) and a history of coronary disease (*p* < 0.0001) have significantly increased. Peripheral arterial disease (*p* = 0.264) and previous carotid disease (*p* = 0.438, not shown in the graph) did not show any differences. The administration of antiplatelet drugs has not been modified (*p* = 0.642), but a progressive increase in the percentage of patients with oral anticoagulation was observed (*p* < 0.0001; 7.2% in 2008 to 13.2% in 2017).

### Trends in mortality, morbidity and good outcome at 3 months

**Fig. 4:**
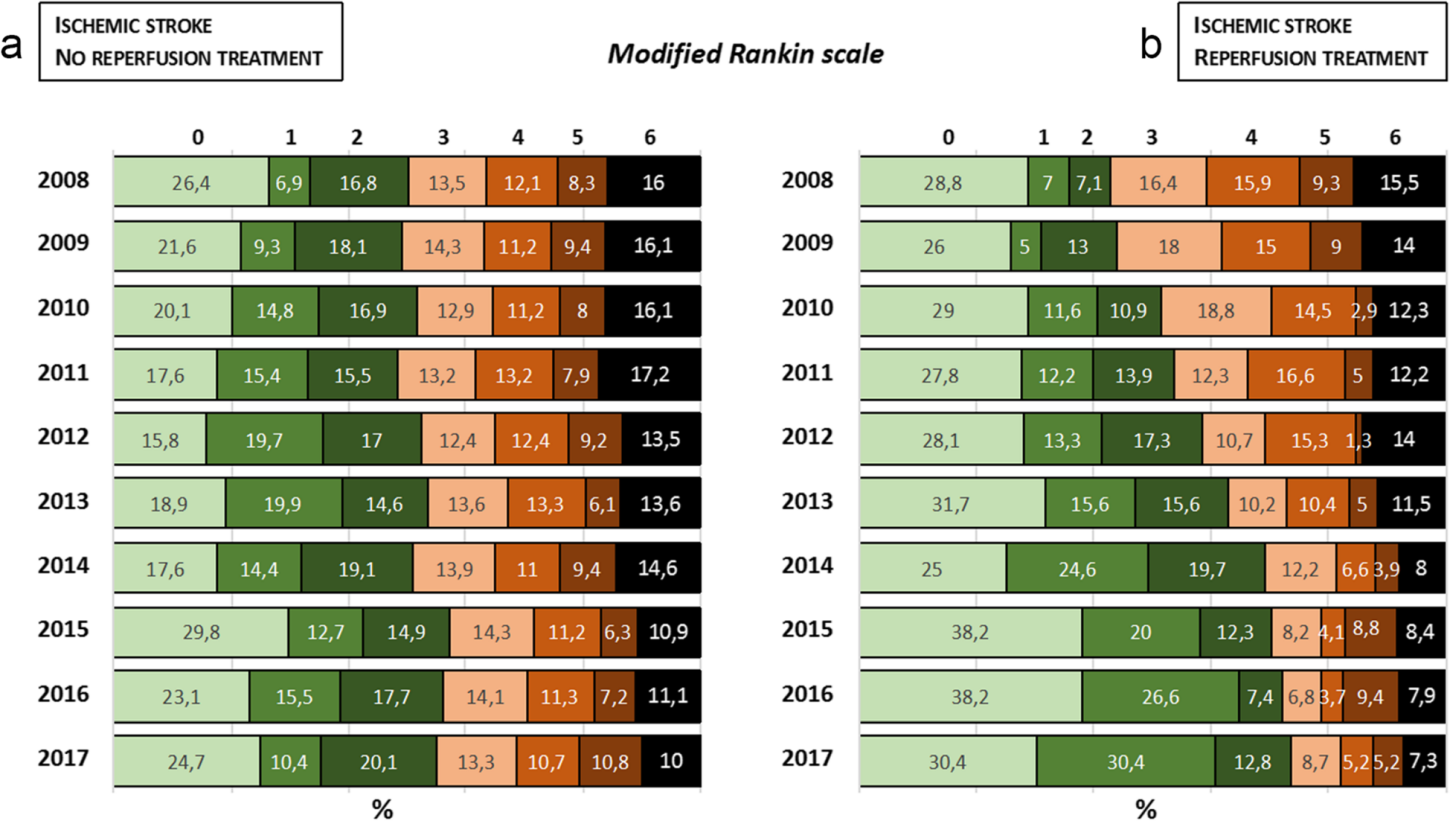
Modified Rankin scale at 3 months: **a** Patients with ischemic stroke without reperfusion treatment. **b** Patients with ischemic stroke with reperfusion treatment

Mortality at 3 months has decreased in all groups of patients (*p* = 0.016). In the IS mortality decreased from 15.4% in 2008 to 9.8% in 2017 (atherothrombotic from 6.0 to 4.7%, cardioembolic from 16.8 to 10.7%, lacunar from 1.6 to 0.8%, undetermined from 10.3 to 6.4%, others from 14.3 to 11.2%). Mortality in ICH decreased from 31.6% in 2008 to 26.6% in 2017. Morbidity at 3 months remained stable in ICH (*p* = 0.709) and IS (*p* = 0.087), except in the subgroup of patients who received reperfusion therapy (*p* < 0.0001). Similarly, all IS groups have also experienced a trend in progress towards a good outcome (*p* = 0.028, 48.8% in 2007 to 55.3% in 2017) with the exception of the ICH (*p* = 0.392). The administration of reperfusion therapy is not only associated with a decrease in mortality, common to all patients with IS, but also with a notable increase in good outcome and a marked decrease in morbidity (Fig. 4a-b).

### Influence of clinical variables on the outcome at 3 months in 2008–2011 and 2012–2017 periods

In the multinomial logistic regression study conducted for the whole sample with ischemic stroke, age, stroke on awakening, higher axillary temperature on admission, baseline blood glucose > 110 mg/dL and a higher white blood cell count were independent factors associated with higher morbidity and mortality. When we analyzed the patients admitted in 2008–2011 and 2012–2017 periods separately, we could see differences in the factors associated with greater morbidity and mortality. The influence of the diagnostic type and the volume of the ischemic lesion has disappeared in recent years (Table 1).

**Table 1:** Multinomial logistic regression: Influence of the clinical, biochemical and neuroimaging variables on the outcome variables (morbidity and mortality) at 3 months from stroke onset

	Ischemic stroke				Intracerebral hemorrhage			
	Morbidity		Mortality		Morbidity		Mortality	
	OR (95% CI)*	*p*	OR (95% CI)*	*p*	OR (95% CI)*	*p*	OR (95% CI)*	*p*
WHOLE SAMPLE								
Age (year)	1.03 (1.02–1.04)	< 0.001	1.06 (1.05–1.07)	< 0.001	1.01 (0.99–1.03)	0.148	1.04 (1.02–1.06)	0.001
Stroke on awakening	1.35 (1.06–1.72)	0.014	1.57 (1.06–2.37)	0.024				
Maximum axillary temperature - first 24 h (**°**C)	1.51 (1.33–1.71)	< 0.001	2.22 (1.06–2.37)	< 0.001	1.32 (0.97–1.79)	0.076	1.92 (1.37–2.68)	< 0.001
Baseline glycemia (mg/dL)	1.00 (1.00–1.00)	< 0.001	1.00 (1.00–1.00)	< 0.001				
Fibrinogen (mg/dL)					1.00 (1.00–1.00)	0.019	1.00 (1.00–1.00)	0.065
White blood cells (×10^3^/mL)	1.11 (1.09–1.14)	< 0.001	1.23 (1.19–1.28)	< 0.001				
Baseline volume of hematoma (mL)					1.03 (1.02–1.04)	< 0.001	1.05 (1.04–1.06)	< 0.001
PERIOD 2008–2011								
Age (years)					1.02 (0.99–1.05)	0.079	1.05 (1.02–1.09)	0.001
Maximum axillary temperature - first 24 h (**°**C)	1.20 (0.86–1.64)	0.280	2.37 (1.47–3.82)	< 0.001				
Baseline glycemia (mg/dL)	1.00 (1.00–1.00)	0.001	1.00 (1.00–1.01)	0.047				
Fibrinogen (mg/dL)					1.00 (1.00–1.00)	0.043	1.00 (1.00–1.00)	0.123
C-reactive protein (mg/L)	1.11 (1.05–1.17)	< 0.001	1.22 (1.14–1.31)	< 0.001				
TOAST:								
- Lacunar	0.29 (0.13–0.64)	0.002	1.14 (0.13–9.90)	0.905				
- Cardioembolic + Indeterminate	1.32 (0.90–1.93)	0.153	3.08 (1.48–6.40)	0.003				
- Atherothrombotic + Others	ref.		ref.					
Ischemic injury volume in 2nd CT (mL)	1.04 (1.03–1.05)	< 0.001	1.05 (1.04–1.06)	< 0.001				
Baseline hematoma volume (mL)					1.03 (1.01–1.05)	0.001	1.08 (1.05–1.10)	< 0.001
PERIOD 2012–2017								
Age (years)	1.04 (1.03–1.05)	< 0.001	1.07 (1.05–1.09)	< 0.001	1.01 (0.97–1.04)	0.679	1.05 (1.01–1.10)	0.025
Maximum axillary temperature - first 24 h (**°**C)	1.12 (1.08–1.15)	< 0.001	1.24 (1.17–1.30)	< 0.001				
Baseline glycemia (mg/dL)	1.00 (1.00–1.00)	0.019	1.00 (1.00–1.00)	< 0.001				
Fibrinogen (mg/dL)					1.00 (1.00–1.01)	0.015	1.00 (0.99–1.00)	0.177
White blood cells (×10^3^/mL)	1.12 (1.08–1.15)	< 0.001	1.24 (1.17–1.30)	< 0.001				
Baseline hematoma volume (mL)					1.03 (1.01–1.06)	0.001	1.05 (1.03–1.07)	< 0.001
Edema volume in 2nd CT (mL)					1.02 (0.98–1.05)	0.290	1.04 (1.00–1.07)	0.047

*Adjusted OR. Good outcome was considered the reference category to calculate the ORs (CI 95%)

In the global population of patients with intracerebral hemorrhage, age and baseline hematoma volume were the only factors associated with greater morbidity and mortality; hyperthermia was associated with mortality and elevated fibrinogen levels with increased morbidity (Table 1). Differentiated multinomial logistic regression analysis in the two time periods did not show differences in the influence of factors associated with higher morbidity and mortality (Table 1).

### Estimation of the forecast trend

**Fig. 5:**
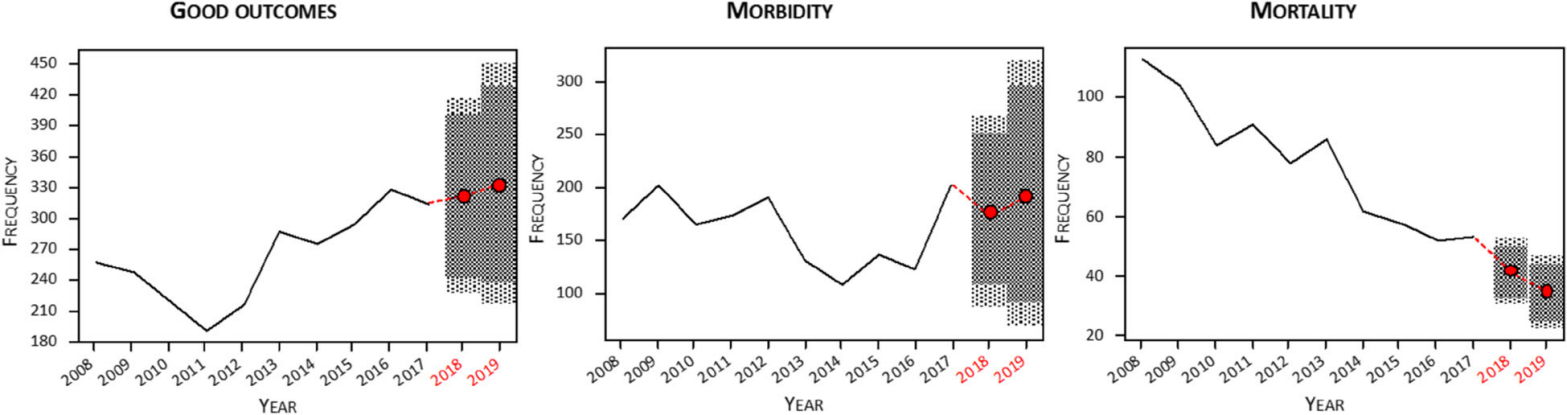
Modeling of time series for good outcome (mRS ≤ 2), morbidity (mRS > 2) and mortality (mRS = 6). The predictions at two years are presented in red, with their confidence intervals at 90% (dark gray) and 95% (light gray)

Time series model allowed us to establish the prognostic progress of patients admitted to our center, given that the current diagnostic and therapeutic procedures have not been substantially modified. In these circumstances, the marked tendency towards decreased mortality (Ljung-Box’s test *p* = 0.2124, Dubin-Watson’s test *p* = 0.7659) can be verified, with a slight increase in good outcome (Ljung-Box’s test *p* = 0.2444, Dubin-Watson’s test *p* = 0.0141), as well as morbidity (Ljung-Box’s test *p* = 0.8284, Dubin-Watson’s test *p* = 0.1007) in the coming years (Fig. 5). Given its statistical weight, this trend is more linked to patients with IS than with intracerebral hemorrhage.

## Discussion

Our results showed remarkable changes in the demography and clinical progress in the 10-year follow-up, which explain the modification in the management of patients with acute cerebrovascular disease. The first modification observed is the rise in the age of stroke presentation that affects women exclusively. This demographic trend may influence comorbidity, the intensity of neurological involvement, access to certain treatments and prognostic evolution. The increase in the incidence of the first stroke, especially in women, has already been confirmed [17, 26–29], in previous literature although it was associated with those geographical areas with a marked aging process [30].

Increase in the age of stroke presentation is likely to be the cause of the greater severity of the neurological symptoms at admission (Spearman coefficient = 0.272; *p* < 0.0001, data not shown in the results), but despite this, a progressive hospital improvement is seen in patients with IS, especially in those who received reperfusion therapies. This trend is not seen in patients with ICH.

The 60-min reduction in the time interval between onset of symptoms and specialized care in the emergency department in the last 10-year is undoubtedly a reflection of the change in the social and health attitude towards stroke. Over the last decade, there has been developed in Spain a specialized press and television advertising campaign about stroke symptoms and ‘time is brain’ concept. The main objective was to inform and sensitize patient’s families and medical services care about the importance of rapidly hospital patient’s transfer. This shortening of the admission time and the speed in healthcare contribute to the rise in the percentage of reperfusion therapies, despite the increase in the age of the population served. The increase in patients treated within the therapeutic window for reperfusion therapies has been the objective achieved by the structural and functional modification developed in the stroke care systems [3, 30–32], although in some cases the total time has not improved [3].

The distribution of the etiological types of intracerebral hemorrhages has remained stable in the last ten years, but in ischemic strokes an increase in cardioembolic strokes has occurred, compensated by a decrease in atherothrombotic ones. This tendency has been reported in other studies in populations with similar characteristics [33–35], but not in younger populations [36].

This increase is not due to an improvement in the identification of embolic sources, since the proportion of undetermined strokes remains stable, but it may be associated with the increase in the age of the population and the greater proportion of elderly women (with more atrial fibrillation). Although the prescription of anticoagulants has increased (from 7.2% in 2008 to 13.2% in 2017), this increase has not been sufficient, since the percentage of patients with atrial fibrillation who do not take anticoagulants has increased (45.0% in 2008 to 58.1% in 2017) but remains significantly below the recommendations of European guidelines [37] and the figures reported for other populations [16].

In contrast to other publications [36], the incidence of risk factors has not changed significantly, with the exception of the decrease in diabetes mellitus and the increase in ischemic heart disease. This paradox could be explained by the marked aging of the population who has had a stroke. The increase in age can explain that the risk factors condition in the first-place coronary disease and that diabetes conditions other vascular diseases in younger ages. The change of attitude in the management of patients with stroke in recent decades has been associated with a progressive trend towards a better prognosis in IS, with a reduction in mortality and improvement of good outcome [18, 29], also found in ICH patients. However, in a meta-analysis that included the 1980–2008 period, the prognosis of ICH remained stable [38].

Reperfusion therapy is the main factor responsible for the change in prognosis and attitude in the management of acute IS [39]. Consequently, we analyzed separately (before and after endovascular treatment was implemented as the standard of care in our hospital in 2012) the factors that could influence the stroke prognosis. In the two time periods, we did not find clear differences of the influence of modifiable factors on morbidity and mortality. The temperature and blood glucose control should condition a decrease or loss of the relationship between these parameters and the prognosis, but we only noted a decrease in temperature and mortality (from an adjusted OR of 2.37 in 2008–2011 to 1.24 in 2012–2017).

Of particular importance for the planning of stroke care in the coming years is the behavior of patients who suffered from significant disabilities (mRS 3, 4 5). In our study, mortality decreases, but the increase in good prognosis is hampered by the tendency to increase morbidity in the coming years. The expected increase in age in the population means that the proportion of patients with ischemic stroke who will not be able to benefit from reperfusion treatment will increase. This rise in morbidity will increase the health expenditure caused by stroke care. The absence of effective treatment for the ICH will aggravate this perspective. It should also be noted that the ICH mortality decrease cannot be associated with a specific factor, however, intensive blood-pressure control, temperature and blood glucose were carried out. Likewise, in recent years the dicumarinics have been replaced by new dependent non-vitamin K anticoagulants (only in a very few cases subjected to clinical trials with anticoagulants).

Our study has some strengths: it encompasses the vast majority of stroke patients treated in a geographical area of half a million inhabitants, with a homogeneous population and minimal immigration. The data were obtained and included by the same neurologists trained in cerebrovascular diseases and all patients were managed under the same protocol. Nevertheless, our study also shows some weaknesses; a relatively short follow-up time (3 months) as a consequence of clinical guidelines. The health area studied belongs to the Atlantic provinces of Galicia consisting mainly of urban and coastal population, but with little rural population. The sample used may be insufficient to establish associations and trends with a strong statistical value. Finally, we consider that the demographic change could make difficult the possible modification of the outcomes of patients in coming years, but future studies should take into account the negative evolution of patients with reperfusion treatment that is not effective [40], the new strategies for combining thrombolytic and endovascular therapies, as well as the analysis of patients from different world regions.

## Conclusion

We found that the age of the stroke patients admitted has increased, especially in women. Hospital improvement has been progressive in the IS, despite a greater severity of patients, and an increase in cardioembolic stroke patients. This trend was not found in patients with ICH. No differences were observed between the incidence of the risk factors and the trend in the poor outcome in the different time periods analyzed. In our study, mortality decreased in the entire sample, but the improvement in the prognosis of patients with IS was negatively compensated by the tendency to an increase in all patients with stroke who had serious sequelae. Our data indicate morbidity is likely to increase in the coming years.

## Abbreviations

AIC: Akaike Information Criteria
AR, MA or ARMA: Autoregressive and moving averages models
CT: Computed tomography
DWI: Diffusion weighted image
EC: Ethics Committee
ICH: Intracerebral hemorrhage
IS: Ischemic stroke
MLR: Multinomial logistic regression
mRS: Modified Rankin scale
NIHSS: National institute of Health Stroke Scale
ProBNP: pro-Brain Natriuretic Peptide
SBIC: Schwarz Information Criteria
TIA: Transient ischemic attack
